# Sources and resources: importance of nutrients, resource allocation, and ecology in microalgal cultivation for lipid accumulation

**DOI:** 10.1007/s00253-014-5694-7

**Published:** 2014-04-03

**Authors:** Matthew W. Fields, Adam Hise, Egan J. Lohman, Tisza Bell, Rob D. Gardner, Luisa Corredor, Karen Moll, Brent M. Peyton, Gregory W. Characklis, Robin Gerlach

**Affiliations:** 1 Department of Microbiology and Immunology, Montana State University, 109 Lewis Hall, Bozeman, MT 59717 USA; 2 Department of Environmental Sciences and Engineering, University of North Carolina, Chapel Hill, NC USA; 3 Center for Biofilm Engineering, Montana State University, Bozeman, MT USA; 4 Department of Chemical and Biological Engineering, Montana State University, Bozeman, MT USA

**Keywords:** Biofuel, Recycle, Algal biofilm, Biofuel ecology

## Abstract

Regardless of current market conditions and availability of conventional petroleum sources, alternatives are needed to circumvent future economic and environmental impacts from continued exploration and harvesting of conventional hydrocarbons. Diatoms and green algae (microalgae) are eukaryotic photoautotrophs that can utilize inorganic carbon (e.g., CO_2_) as a carbon source and sunlight as an energy source, and many microalgae can store carbon and energy in the form of neutral lipids. In addition to accumulating useful precursors for biofuels and chemical feed stocks, the use of autotrophic microorganisms can further contribute to reduced CO_2_ emissions through utilization of atmospheric CO_2_. Because of the inherent connection between carbon, nitrogen, and phosphorus in biological systems, macronutrient deprivation has been proven to significantly enhance lipid accumulation in different diatom and algae species. However, much work is needed to understand the link between carbon, nitrogen, and phosphorus in controlling resource allocation at different levels of biological resolution (cellular versus ecological). An improved understanding of the relationship between the effects of N, P, and micronutrient availability on carbon resource allocation (cell growth versus lipid storage) in microalgae is needed in conjunction with life cycle analysis. This mini-review will briefly discuss the current literature on the use of nutrient deprivation and other conditions to control and optimize microalgal growth in the context of cell and lipid accumulation for scale-up processes.

## Introduction

In modern societies, petroleum-based products and fuels have strongly influenced human culture and infrastructure. For example, energy, food, and chemicals make up approximately 70 % of commerce on the planet (www.eia.gov), and petroleum/hydrocarbons directly and indirectly impact these commodities. Petroleum/hydrocarbon markets have become increasingly unpredictable and cause destabilized commodity prices (e.g., fuel, food). In addition, the environmental impacts from increased carbon dioxide (CO_2_) without balanced CO_2_ sequestration has contributed to increases in atmospheric CO_2_ levels. The amount of carbon released in 1 year from the consumption of fossil fuels is more than 400-fold the amount of carbon that can be fixed via net global primary productivity (Dukes 2003). In order to offset the massive influx of CO_2_ into the atmosphere, the utilization of renewable biofuels (e.g., ethanol, butanol, H_2_, CH_4_, and biodiesel) is needed.

Bacillariophyta (diatoms) and Chlorophyta (green algae) are eukaryotic photoautotrophs that can utilize inorganic carbon (e.g., CO_2_) as a carbon source and sunlight as an energy source, and many microalgae can store carbon and energy in the form of neutral lipids [e.g., triacylglycerides (TAGs)]. Moreover, different diatoms and algae can produce and accumulate different precursors (e.g., carbohydrates, fatty acids, and pigments) that are value-added products. In addition to accumulating useful compounds for biofuels and chemical feed stocks, the use of autotrophic microorganisms can further contribute to reduced CO_2_ emissions through utilization of atmospheric CO_2_. For these reasons, eukaryotic photoautotrophs have been studied in the context of lipid accumulation for over 50 years and were a focus of the US Department of Energy’s Aquatic Species Program in the 1980s and 1990s (Sheehan 1998). However, low petroleum prices eventually eroded monetary support for alternative (and renewable) energy sources until increasing petroleum prices over the last two decades reinvigorated interest in alternatives.

The advent and increased use of fracking technologies has opened up new petroleum and hydrocarbon reservoirs, and almost $190 × 10^9^ was spent in the USA in 2012 to drill and “frac” for conventional hydrocarbons (www.eia.gov). However, the process of fracking increases the production rate and not the ultimate supply of hydrocarbons, and peak hydrocarbon production is predicted to occur around 2030 (www.eia.gov). Regardless of current market conditions and availability of conventional sources, alternatives are needed to circumvent future economic and environmental impacts from continued exploration and harvesting of conventional hydrocarbons.

Conservative estimates predict (assuming a lipid content of 25–30 % (*w*/*w*) in microalgae) that an area equivalent to 3 % of the arable cropland in the USA would be required to grow sufficient microalgae to replace 50 % of the transportation fuel needs in the USA (Chisti 2007; Georgianna and Mayfield 2012). Although the interest in algal biofuels has been reinvigorated (Courchesne et al. 2009; Greenwell et al. 2010; Razghefard 2013), significant fundamental and applied research is still needed to fully maximize algal biomass and biochemical production for biofuels and other products.

**Fig. 1:**
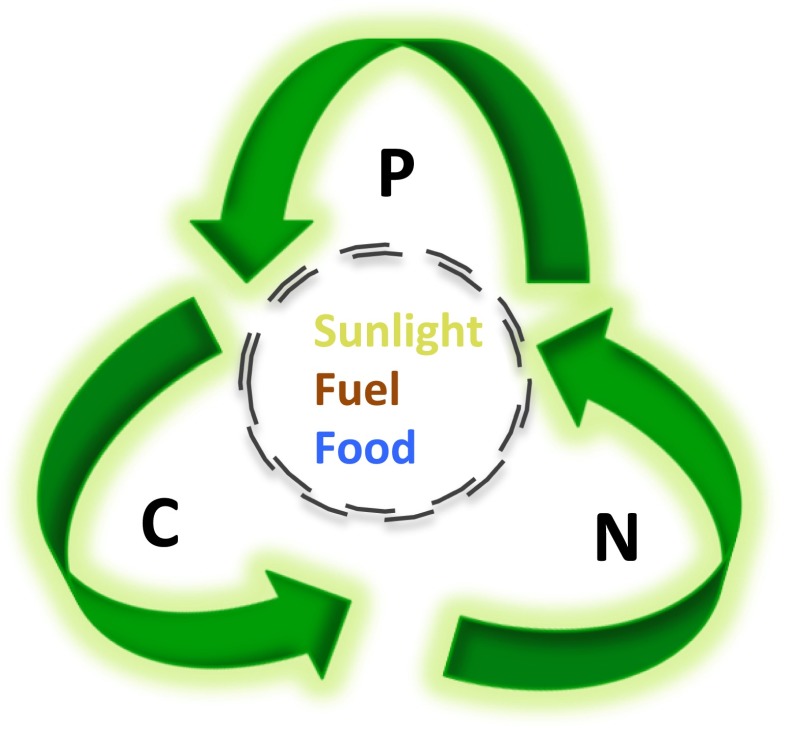
The biological recycling of carbon, nitrogen, and phosphorus to harvest fuel and food linked to sunlight to reduce net consumption of N and P and net production of C

The accumulation of lipids is of substantial interest because these compounds are energy-rich biodiesel precursors (Dismukes et al. 2008; Hu et al. 2008). Much of the reported research has focused on increasing algal lipid accumulation upon exposing cultures to a range of environmental stresses prior to harvest (Hu et al. 2008; Valenzuela et al. 2012, 2013; Mus et al. 2013; Lohman et al. 2013; references therein). Temperature variations, pH, salinity, light, and osmotic and chemical stress inducements have also been investigated with varying success (Sharma et al. 2012). While a stress event can increase lipid accumulation, it can also limit biomass production, but the stress scenario provides a tractable method to study and understand lipid accumulation at the laboratory scale (Valenzuela et al. 2013). Because of the inherent connection between carbon (C), nitrogen (N), and phosphorus (P) in biological systems, macronutrient deprivation has been proven to significantly enhance lipid accumulation in different diatom and algae species. While nitrogen limitation is the most commonly studied stress in green algae and diatoms; the effect of silica limitation is regularly studied in diatoms (Valenzuela et al. 2012; Lohman et al. 2013; Chu et al. 2013; Schnurr et al. 2013). Light and temperature are also known stressors that can impact lipid accumulation (Hu et al. 2008), and particular wavelengths have been shown to impact the rate and amount of accumulated lipid in *Chlorella* (Atta et al. 2013). Keeping in mind that a vast majority of living pools of C, N, and P resides in the microbial realm (Whitman et al. 1998), much work is needed to understand the link between C, N, and P in controlling resource allocation both with respect to natural and man-made systems. In this context, a 50 % replacement of transportation fuel by renewable biological sources would impose a vast nutrient demand (Pate et al. 2011). However, microalgal biomass/product production can be coupled to wastewater resources (e.g., water, N, and P), and wastewater from agricultural, industrial, and municipal activity may provide a cost-effective source of nutrients. Agricultural and municipal wastewater can be high in N and P (Aslan and Kapdan 2006; Hoffmann 2002; Mallick 2002; Pittman et al. 2011), and thus, there is a great potential for the integration of wastewater treatment and algal biofuel/biomass production (Fig. 1). However, an improved understanding of the relationship between the effects of N, P, and micronutrient availability on cellular resource allocation (cell growth versus lipid storage) in microalgae is needed. This mini-review will briefly discuss the current literature on the use of nutrient deprivation and other conditions to control and optimize microalgal culture growth in the context of cell and lipid accumulation.

### Nutrient-dependent lipid accumulation

**Fig. 2:**
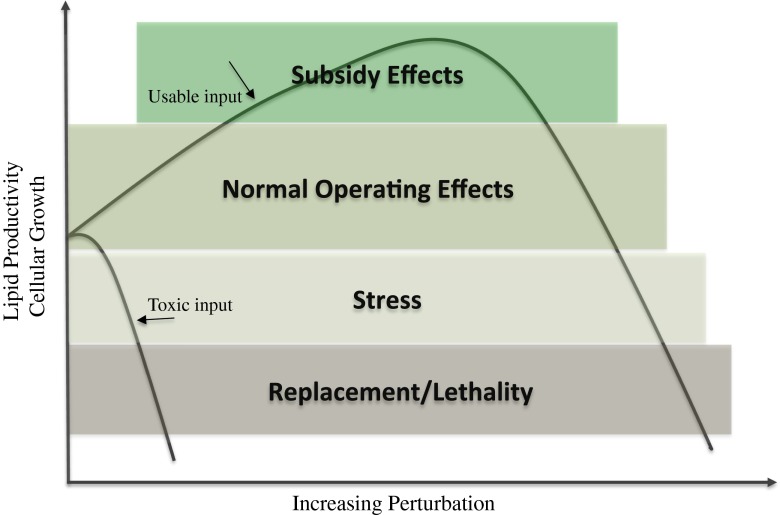
Hypothetical performance curve for an increasingly perturbed (i.e., stressed) microalgal system being used to produce photoautotrophic biomass and/or lipids. Adapted from Odum et al. (1979)

Under optimal growth conditions, (i.e., adequate supply of nutrients including C, N, P, and sunlight), algal biomass productivity can exceed 30 g dry weight per square meter per day (Gordon and Polle 2007); however, the lipid content of the biomass is typically very low (<5 % *w*/*w*) and is species-dependent (Gordon and Polle 2007). The low-lipid content is due to lipid biosynthesis being a metabolic process that is typically stimulated by stress inducement. Essentially, biomass synthesis and lipid biosynthesis compete for photosynthetic assimilation of inorganic carbon, and a fundamental metabolic switch is required to shift from biomass production to energy storage metabolism (Schuhmann et al. 2012; Valenzuela et al. 2012). As denoted by Odum (1985), stress is a syndrome that consists of inputs and outputs, and the input is the stressor that is contrasted to the stress, or the output. Lipids (the output) are typically believed to provide a storage function within the cell that enables the organism to endure adverse environmental conditions, i.e., the stressor. The output can be viewed as the cessation of cell production and the accumulation of lipids in response to the input of unbalanced resources (e.g., N, P, and/or sunlight). It is likely that there are tradeoffs in terms of biomass versus lipid accumulation depending on the different levels of perturbation (Fig. 2).

Recent research has provided evidence that lipids may also act as a reservoir for specific fatty acids such as polyunsaturated fatty acids (PUFAs) (Ratha et al. 2013). PUFAs play a key role in the structural components of cell membranes, and as antioxidants (PUFAs can counteract free radical formation during photosynthesis). As such, PUFA-rich TAGs might donate specific compounds necessary to rapidly reorganize membranes through adaptive metabolic responses to sudden changes in environmental conditions (Khozin-Goldberg and Cohen 2006). However, a recent study showed that PUFA content in lipids can negatively impact biodiesel quality based upon lipids from *Chlorella pyrenoidosa* (Shekh et al. 2013), and this result suggests that lipid composition, and not just amounts, should be considered. In either case, lipid is an energy-rich storage compound that can be chemically transesterified to produce fatty acid methyl esters (FAME), the biological equivalent to diesel fuel (a.k.a., biodiesel). However, to maximize lipid biosynthesis, the producing organism is typically induced through environmental stress conditions (Hu et al. 2008). In addition, most studies have been based upon axenic cultures with limited understanding of potential bacterial “contamination,” and thus, lipid accumulation may be different at different scales of biological resolution (discussed below).

Significant work has been done to identify and optimize stress-inducement strategies that enhance lipid accumulation in microalgal species. Nutrient deprivation, specifically nitrogen depletion, is the most prevalent technique employed (Hu et al. 2008). This may be due to two factors: (1) Lack of requisite nutrients such as nitrogen limits the capacity to synthesize proteins necessary for biomass production (e.g., cellular division). In order to compensate, the organism must take advantage of alternative metabolic pathways for inorganic carbon fixation, such as fatty acid synthesis and hence, store those de novo fatty acids as TAG (Msanne et al. 2012). (2) Photosynthesis and the electron transport chain in eukaryotic microalgae produce ATP and NADPH as energy “storage” and electron carrier metabolites, respectively (Halsey et al. 2013). These metabolites are consumed during biomass production resulting in ADP and NADP^+^, which in turn, are regenerated via photosystems. Under normal growth conditions, this cycle maintains a balanced ratio of the reduced and oxidized forms of these metabolites; however, when biomass production is impaired due to a lack of requisite nutrients, the pool of NADP^+^ and ADP can become depleted. This can lead to a potentially dangerous situation for the cell because photosynthesis is mainly controlled by light availability, and cannot be shut off completely. Fatty acid synthesis consumes NADPH and ATP; therefore, increased fatty acid synthesis replenishes the pool of required electron acceptors in the form of NADP^+^, and de novo fatty acids are most frequently stored as lipid (Brown et al. 2009). Here, we will review the most successful strategies involving nutrient stress to induce lipid accumulation in commonly studied microalgal species.

### Nitrogen and phosphorus

Nutrient availability is critical for cell division and intracellular metabolite cycling, and once nutrients such as N or P become depleted or limited in the medium, invariably, a steady decline in cellular reproduction rate ensues. Once this occurs, the activated metabolic pathways responsible for biomass production are down-regulated and cells instead divert and deposit much of the available C into lipid (Wang et al. 2009; Valenzuela et al. 2013). There have been numerous studies to compare different N sources in the context of maximal biomass or lipid accumulation, and the results are different dependent upon the organism. Breuer et al. (2012) accumulated previous literature on 56 eukaryotic, photoautotrophic genera studied in the context of lipid accumulation (Table 1). The authors chose *Chlorella vulgaris*, *Chlorella zofingiensis*, *Nannochloris* UTEX 1999, *Neochloris oleoabundans*, *Scenedesmus obliquus*, *Dunaliella tertiolecta*, *Isochrysis galbana*, *Phaeodactylum tricornutum*, and *Prophyridium cruentum* to conduct normalized growth and lipid accumulation studies with nitrate as the N source (Breuer et al. 2012). Under N deprivation, *Chlorella vulgaris*, *Chlorella zofingiensis*, *Neochloris oleoabundans*, and *Scenedesmus obliquus* accumulated over 35 % dry weight as TAG, and *Scenedesmus obliquus* and *Chlorella zofingiensis* had the highest TAG productivity (240–320 mg L^−1^ day^−1^) among the nine compared strains.

**Table 1:** Genera of 56 eukaryotic, photoautotrophs previously studied and reported for the accumulation of lipids. Modified from Breuer et al. (2012)

*Amphora*	*Ankitrodesmus*	*Biddulphia*	*Botryococcus*
*Bracteacoccus*	*Chaetoceros*	*Chlamydomonas*	*Chlorella*
*Chlorococcum*	*Chroomonas*	*Cryphecodinium*	*Cryptomonas*
*Cylindrotheca*	*Dictyosphaerium*	*Dunaliella*	*Ellipsoidion*
*Emuliania*	*Enteromorpha*	*Euglena*	*Fragilaria*
*Glossomastrix*	*Gymnodinium*	*Haematococcus*	*Hantzchi*
*Hemiselmis*	*Isochrysis*	*Monallantus*	*Monodus*
*Nannochloris*	*Nannochloropsis*	*Navicula*	*Neochloris*
*Nephroselmis*	*Nitzschia*	*Ochromonas*	*Parietochloris*
*Pavlova*	*Phaeodactylum*	*Pheomonas*	*Polytoma*
*Porphyridium*	*Protosiphon*	*Prototheca*	*Rhodomonas*
*Rhodosorus*	*Scenedesmus*	*Scrippsiella*	*Selenastrum*
*Skeletonema*	*Stichococcus*	*Tetraselmis*	*Thalassiosira*
*Ulothirx*	*Volvox*		

When the model Chlorophyte *Chlamydomonas reinhardtii* was cultivated under N limitation, an increase in lipid was also observed. Interestingly, fully saturated C_16_ fatty acids were the most abundantly synthesized compounds, whereas polyunsaturated C_18_ fatty acids remained relatively unchanged in this organism under the tested conditions (Lohman et al. 2013). While nitrate supported increased biomass compared to ammonium in *Monoraphidium* sp. SB2 (Wu et al. 2013), *Chlorococcum ellipsoideum* exhibited elevated lipid levels with urea compared to nitrate (Li et al. 2013). A different *Scenedesmus* strain (sp. R-16) was shown to have the highest lipid accumulation with nitrate compared to urea, peptone, or yeast extract (Ren et al. 2013). To date, nitrate is a commonly studied N source used to understand nutrient deprivation to induce lipid accumulation; however, different N sources have different effects dependent upon the organism. This is most likely a consequence of typical habitat for the organism as well as long-term life history that is common for the respective species. As the need for nutrient recycling becomes more evident, different types and mixtures of nutrients (e.g., human, agriculture, industrial) must continue to be evaluated. For example, two recent studies investigated the ability of *Chlamydomonas polypyrenoideum* and *Chlorella pyrenoidosa* to grow and accumulate lipids during cultivation on dairy wastewater (Kothari et al. 2012, 2013), and we recently grew a green alga isolated from storage ponds of coal-bed water that produced lipids under nutrient deprivation (Fields, Nagy, and Barnhart, unpublished results). Nitrogen deprivation was shown to induce lipid accumulation in the wastewater isolates, *Scenedesmus* sp. 131 and *Monoraphidium* sp. 92 with ammonium, nitrate, or urea (Eustance et al. 2013) or nitrate depletion in *Skeletonema marinoi* (Bertozzini et al. 2013). Interestingly, *Ettlia oleoabundans* initiated lipid accumulation in response to increased temperature before nitrate was completely depleted (Yang et al. 2013). These results suggest that different combinations of potential stressors could impact lipid accumulation in different ways.

In addition to N, P starvation to induce lipid accumulation in microalgae has been studied as a sole stress or in combination with N limitation. In general, greater lipid accumulation due to N deprivation has been observed compared to P deprivation as reported for various *Chlorella* species (Feng et al. 2012; Liang et al. 2012). When the marine diatom *Phaeodactylum tricornutum* was grown under N and P limitation, an increase in lipid accumulation was noticed in all limiting conditions (Valenzuela et al. 2012; Burrows et al. 2012). However, cultures of *Phaeodactylum tricornutum* that were limited exclusively in N showed a more significant increase in TAG than cultures that were limited solely in P. The combined limitation of both N and P resulted in the highest lipid concentrations in *Phaeodactylum tricornutum* (Valenzuela et al. 2012, 2013). Given the commonly accepted N/P ratio of 16:1 in microalgal biomass (Redfield 1958), the *Phaeodactylum tricornutum* work demonstrated that the external N/P ratio was 27 and the cellular N/P ratio was between 8:1 and 9:1 when lipid accumulation was observed (Valenzuela et al. 2012).

Both N and P deprivation result in cell cycle cessation, but the relative lipid accumulation response is different, and this observation is most likely a consequence of cellular resource allocation (e.g., protein/chlorophyll vs. nucleotides). Based upon results in *Phaeodactylum tricornutum*, we observed a fivefold greater increase in specific fluorescence of Nile Red, a commonly used indicator of lipid accumulation (Gardner et al. 2011), when cells were depleted of nitrate compared to cells depleted of phosphate. In addition, resupplementation of N or P promoted cellular growth, cessation of lipid accumulation, and increased lipid consumption in *Phaeodactylum tricornutum* (Valenzuela et al. 2013).

### Carbon

It is important to keep in mind that when comparing different nutrient-deprived states, carbon, above all else, is absolutely required for lipid biosynthesis (Palmqvist et al. 1988; Spalding 2008). Without carbon, independent of nutrient deprivation, biomass or lipid biosynthesis is impossible. Therefore, the most successful reports of lipid induction techniques in microalgal lipid production typically involve elevated concentrations of inorganic carbon in tandem with N and/or P limitation (Gardner et al. 2011, 2012; Sharma et al. 2012). These strategies often employ a CO_2_ sparge to increase dissolved CO_2_ above atmospheric concentrations, or addition of soluble inorganic carbon during inoculation or just prior to nutrient depletion (Gardner et al. 2011, 2012). It should be kept in mind that the addition of soluble inorganic carbon (e.g., bicarbonate) can also affect pH and osmolarity. The addition of large amounts of dissolved inorganic carbon via a CO_2_ gas sparge can contribute significantly to the production cost in an algal biorefinery (e.g., Liu et al. 2013), and alternative methods to gaseous CO_2_-based carbon supply should be considered in conjunction with pH control. Gardner et al. (2011, 2012) demonstrated that the dosage of small amounts of bicarbonate, solely or in combination with a CO_2_ sparge, can achieve similar algal growth and lipid production yields compared to continuous CO_2_ sparging. The use of bicarbonate addition, versus CO_2_ sparging, could result in significantly lower equipment costs. In either case, elevated concentrations of C, combined with N or other nutrient deprivation, has been shown to induce lipid accumulation in virtually every microalgal species tested. However, an improved understanding of cellular and population responses to not only the respective concentrations but the ratios of macronutrients (e.g., C, N, and P) will improve resource utilization and promote efficient, cost-effective processes.

### Silicon limitation

Reports on silicon limitation have revealed that both marine and freshwater diatoms will accumulate lipid under Si-limiting conditions (Sharma et al. 2012), and diatoms possess immense potential as contributors to biodiesel production. When faced with Si-limitations, most diatoms appear to direct carbon storage towards lipid (Roessler 1988), albeit the response is dependent on the degree of Si content in the cell wall. Diatoms incorporate biologically available Si as monomeric or dimeric silicic acid into silicious cell walls (frustules) and require approximately 7 % of the energy expenditure required for polysaccharide cell wall formation characteristic of green algae (Hildebrand et al. 2012; Kroger and Poulsen 2008; Raven 1983). Diatoms produce comparatively less cellular starch, such that fixed carbon has increased potential to be allocated to lipid accumulation (Burrows et al. 2012; Gardner et al. 2011; Roessler 1988; Smith et al. 2012). In fact, diatom cells can accumulate enough TAG to cause the frustules to break under silica deplete conditions (Hildebrand et al. 2012), potentially reducing the need for energy intensive procedures associated with lipid extraction in green algae.

Numerous studies have shown increased lipid accumulation when diatoms are cultured in silica deplete media (Lombardi and Wangersky 1991; McGinnis et al. 1997; Obata et al. 2013; Yu et al. 2009). However, the majority of these studies were performed on marine diatoms (e.g., *Cylindrotheca spp*., *Thalassiosira pseudonana*, and *Phaeodactylum tricornutum*) grown in media containing comparatively lower silica concentrations (Taguchi et al. 1987; Roessler 1988; Yu et al. 2009). The results of Moll et al. (2014) indicate that increasing the silica concentration will increase cell numbers, which is vital for improving algal biodiesel productivity in terms of increased biomass. Therefore, while research on marine diatoms for use in biofuel applications may be advantageous for use in large-scale raceway ponds due to the ability to tolerate saline environments, the actual use may be limited until conditions are optimized for diatom cell growth and lipid accumulation.

While silica limitation is known to increase lipid accumulation, combined with other physiological stresses, lipid accumulation may be enhanced. A recent study investigated the effect of coincident silica and nitrate limitation and HCO_3_^−^ addition to promote lipid accumulation in a freshwater diatom. Moll et al. (2014) observed that combined silica and nitrate limitation, as well as sodium bicarbonate addition increased lipid accumulation compared to individual stressors with or without HCO_3_^−^. One hypothesis for this observation is the effect on the cell cycle. Olson et al. (1986) and Vaulot et al. (1987) revealed that for *Thalassiosira weisflogii* and *Hymenomonas carterae*, nitrate and silica limitation resulted in halting the cell cycle at G_1_ and the G_1_/S and G_2_/M boundaries, respectively (Darley and Volcani 1969). It is possible that the two combined nutrient limitations at different periods within the cell cycle may contribute to cellular stress and ultimately lead to enhanced lipid accumulation in diatoms.

### Iron limitation

As mentioned above, N, P, and C are the most important macronutrients, but Fe is the most versatile and important trace element for biochemical catalysis. Approximately 30 to 40 % of the world’s oceans are iron limited, and studies have investigated “iron fertilization” experiments whereby iron is added to high-nutrient low chlorophyll (HNLC) areas to induce phytoplankton growth and CO_2_ fixation (Buesseler et al. 2004). Iron-limited conditions are thought to alter cell physiology by reducing cell volume, chlorophyll content, and photosynthetic activity, and thus appear to impact cellular accumulation more than lipid accumulation per se. Specifically in *Phaeodactylum tricornutum*, the following enzymes were down-regulated during iron-starvation: β-carbonic anhydrase, phosphoribulokinase (PRK), two RuBisCO enzymes and a HCO_3_^−^ transporter, likely resulting in decreased carbon fixation and cellular growth (Allen et al. 2008). The results suggest that iron limitation greatly impacts cell growth and accumulation, and that approximately 10 μmol Fe/mol C is needed by marine algae (Morel et al. 1991). Iron limitation has also been linked to increased rates of silicification, thus increasing cell density and cell sinking. According to Allen et al. (2008), cells grown under Fe-limited conditions fixed carbon 14 times slower compared to cells grown in iron-replete conditions. Since iron limitation can result in detrimental physiological effects, it is pertinent to determine the potential for these processes to be useful for commercial scale lipid accumulation.

### Biofilm growth

One of the most significant limitations to the economical use of algae is the high cost of harvesting and concentrating the biomass (Johnson and Wen 2010; Christenson and Sims 2012; Ozkan et al. 2012; Schnurr et al. 2013; Bernstein et al. 2014). To date, research has been focused on microalgae in suspended phase for lipid production, and few studies have focused on the biofilm growth state. However, the biofilm growth state provides some advantages over suspended growth systems in terms of biomass accumulation and maintenance that would be beneficial for biomass harvesting and concentrating prior to processing. Algal suspensions are often between 0.02 and 0.06 % total suspended solids (TSS), and significant energy is required to harvest and concentrate the cells to 5 to 25 % TSS. Biofilms can range from 6 to 16 % TSS (Schnurr et al. 2013), and could potentially minimize biomass-processing costs (Johnson and Wen 2010; Christenson and Sims 2012; Ozkan et al. 2012). In general, the available algal biofilm studies are based upon wastewater treatment, biofilm structure and development, and aquaculture applications (Johnson and Wen 2010; Christenson and Sims 2012; Patil and Anil 2005; Irving and Allen 2011; Avendaño-Herrera and Riquelme 2007). There is a small amount of research on biofilm systems for the production of biomass and lipids in eukaryotic photoautotrophs (Schnurr et al. 2013; Bernstein et al. 2014), but very little in relation to the influence of environmental stresses.

Recently, Schnurr et al. (2013) reported biofilm growth under nutrient starvation to stimulate lipid accumulation. A semi-continuous flat plate parallel horizontal photobioreactor system (PBR) was designed to control the bulk medium nitrogen and silicon concentrations until nutrient depletion and biofilm onset. Wastewater was used to seed biofilm growth and was later replaced by synthetic medium and pure cultures of *Nitzschia palea* and *Scenedesmus obliquus*. Well-attached, thick algal biofilms were observed in all experiments, until N and Si levels decreased to below detection limits, resulting in detachment from the substratum. In contrast to suspended algae, the algal biofilms did not accumulate more neutral lipids when exposed to nutrient-deficient conditions in these studies. Similar results were reported by Bernstein et al. (2014) who observed little lipid accumulation in mixed culture wastewater biofilms on the field scale or in laboratory-scale algal biofilm reactors seeded with a *Botryococcus* sp. (strain WC2B). Based upon these results, there appears to be fundamental differences in the way suspended cultures and biofilm cultures respond to nutrient deprivation. The exact reasons for differences between suspended and biofilm cells are unknown, but may be a consequence of altered nutrient cycling in biofilms due to altered carbon flow for cellular turnover and compound accumulation. A result of “community growth” (i.e., biofilms) may be to accumulate excess C and reducing equivalent as cells and exopolymer rather than internal storage molecules (e.g., lipid). Future work is needed to discern the differences between the physiological states of biofilm and free-living cells in multiple species.

It is possible that benthic microorganisms would prove more useful for biofilm growth modes, and we have recently grown a benthic diatom in biofilm reactors that could accumulate lipids (Fields, Whitney, and Valenzuela, unpublished results). These results suggest that the two growth modes can elicit different behaviors, and numerous research approaches and questions need to be explored to better understand the feasibility and cellular responses of microalgal biofilms for biomass and lipid accumulation.

### Ecological effects

The literature offers many examples of increased lipid production in numerous algal species cultivated as monocultures in closed photobioreactor (PBR) or open raceway systems under varying nutrient limitations. However, as demonstrated by mathematical models and field experiments, phytoplankton biodiversity can be correlated to increased productivity (Tilman 1981; Downing and Leibold 2002; Striebel et al. 2009). Furthermore, in natural freshwater systems, productivity, measured as biomass, was highest when there was abundant nutrient availability (Interlandi and Kilham 2001). These observations underlie the challenge of needing high biomass loads to maximize overall lipid production. Obviously, productivity can fall under the guise of several metrics ranging from biomass, cell number, chlorophyll/pigments, and more recently, lipid accumulation. Despite the success of increased lipid content in nutrient-deprived monocultures, recent studies indicate that comparable lipid production can also be achieved in nutrient-rich systems with a diverse community.

A study by Stockenreiter et al. (2012) demonstrated increased lipid production in naturally occurring algal communities compared to that of single species cultivated in PBRs (Stockenreiter et al. 2012). For freshwater systems, P is the key nutrient responsible for eutrophication and can greatly alter productivity when limited (Schindler 1977; Smith and Bennett 1999; Schindler et al. 2008; Stockenreiter et al. 2012). Based upon observations with PBRs with deplete nutrient availability, one would expect to see substantially higher lipid content in the oligotrophic communities than the eutrophic. However, Stockenreiter et al. (2012) showed a linear increase in total algal lipid content in correlation with species richness of the examined communities and that lipid content in communities did not differ significantly from 22 laboratory monocultures (1.4 × 10^6^ versus 3.3 × 10^6^ pg ml^−1^). Although in no way conclusive, results such as these suggest comparable lipid values in nutrient replete and deplete systems and indicate the need to further investigate the relationship between nutrient type and abundance in the context of lipid production in mixed communities (Stockenreiter et al. 2013).

A diverse community is also more resistant to invasion from other species that could outcompete the desired algal species (Tilman 1977, 1981). Higher nutrient availability may also aid in algal cultivation by making algae less susceptible to viral infection. Coupled with community diversity, the relative health of each species is an important component. “Healthy” algae (i.e., cells not under nutrient stress) can be more resistant to viral infection that leads to cell lysis (Murray 1995). Rhodes and Martin (2010) developed a theoretical model that implicated high-nutrient availability in significantly reduced viral infection, and such scenarios will be important to consider in microalgal cultivation processes.

In contrast, despite the success of increased lipid accumulation in PBR monocultures under nutrient-limiting conditions, economic assessments indicate that PBRs operating on a large scale may not be commercially viable (Smith 2009). However, some have argued for hybrid systems that utilize a combination of both closed and open systems (Singh and Dhar 2011), or modified PBRs such as solid-state reactors (Naumann et al. 2013). In addition, if ponds are not well mixed, biomass loss due to dark respiration may impact performance for some microalgae (Huesemann et al. 2013). The ecology of open and closed systems will have different parameters and inputs that need to be considered in order to control and optimize ecosystem function (e.g., biomass, lipids, high value compounds). Therefore, life cycle analyses should help direct research to identify complementarity between water footprint, nutrient sources, regional light availability, process design, and targeted lipid-producing organisms.

### Integrating life cycle analysis

**Fig. 3:**
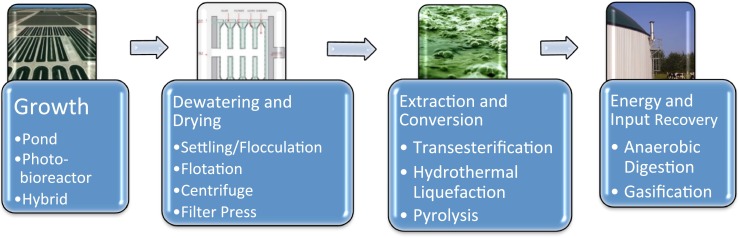
Primary stages and (alternative processes) in the microalgae to fuel production process

Algal biofuels have the potential to provide a substantial fraction of US transportation fuel while imposing a relatively small (arable) land footprint (Sander and Murthy 2010) and providing opportunities for reducing water and nutrient consumption relative to first generation biofuels (Clarens et al. 2011). The degree to which biology and engineering can contribute to these goals will, however, be a function of the entire life cycle (Zaimes and Khanna 2013). A circumscribed version of that life cycle consistent with this review, one involving only the production cycle (distinct from the usage cycle), includes microbial growth, dewatering/drying, extraction/conversion energy/input recovery stages, with each stage involving a number of choices (Fig. 3).

With respect to benefits, life cycle analysis (LCA) has promoted system optimization by highlighting processing alternatives that produce a net increase in system performance, while also avoiding environmental “burden shifting” that can be obscured when viewing the production system less holistically (Klöpffer 1997). In addition, the growth stage will be significantly affected by choices made in the other three production stages, with the extraction/conversion technology having particular importance (Brennan and Owende 2010). While each has respective strengths and weaknesses, two of the most critical distinctions from a life cycle perspective are (a) the degree of pre-conversion drying required (Lardon et al. 2009) and (b) whether the conversion process involves all of the algal biomass or only the lipid fraction (Kirrolia et al. 2013).

The dependence of transesterification processes on algal lipid content can impose extra costs in the growth stage (Brennan and Owende 2010), and lipid accumulation procedures typically come at the cost of algal productivity (Davis et al. 2011). As noted by Quinn et al. (2013) and Chowdhury et al. (2012), increasing lipid content can result in an increase in processing greenhouse gas (GHG) emissions, because less residual biomass is used in a potential energy recovery stage. Thus, the grid energy requirement increases proportionally with the lipid fraction. Wet extraction transesterification processes, while significantly reducing the drying energy input, typically involve solvent-based extractions that lead to concerns over solvent disposal (Torres et al. 2013). In addition, solvent recycling can be challenging and energy intensive due to the high volumes and accompanying wet slurry (Ríos et al. 2013). Simultaneous extraction and transesterification processes (i.e., “reactive extraction”) offer the potential for increased oil yields and lower process costs (Rawat et al. 2013), but the effectiveness of these processes at an industrial scale is still untested (Nagarajan et al. 2013).

Hydrothermal liquefaction (HTL) processes, despite greater capital expense, also reduce drying/dewatering requirements through the utilization of a wet feedstock (López Barreiro et al. 2013), while converting up to 60 % of the total biomass into a useable fuel (Liu et al. 2013). As a result, HTL can increase fuel yields relative to transesterification (Frank et al. 2013), and this technology may reduce the importance of advanced culturing methods to enhance algal lipid accumulation for biofuel production (Elliott et al. 2013). However, thermochemical conversion methods such as HTL make nutrient recycling less efficient, as the nutrient-rich byproducts are poorly suited for direct recycling into the growth process or anaerobic digestion (López Barreiro et al. 2013). In addition, N loss during the conversion process results in a substantially increased nutrient requirement in the growth stage (Liu et al. 2013).

When attempting to assess the economic competitiveness of algal biofuels, an important source of uncertainty in existing studies is the extrapolation of lab and pilot-scale data to industrial scale production (Collet et al. 2013). The use of harmonization procedures (Sun et al. 2011) to reduce variability between multiple studies has allowed for more direct comparison of similar production frameworks given different assumptions. By harmonizing results from several sources, the base cost of production converged to $11.57 gal^−1^ with a standard deviation of $1.17 gal^−1^, this from a pre-harmonization value of $19.10 gal^−1^ and a standard deviation of $6.22 gal^−1^ (Slade and Bauen 2013). Given energy content, algae are predicted to require a production cost of $2.83 gal^−1^ to be competitive with petroleum-based fuels (based on March 2014 oil prices) (Chisti 2007). However, many technological advances must occur to reach this point, and one issue that has received significant attention is the choice of microbial growth technology.

A wide range of different, and often contradicting, results have arisen from economic comparisons of microbial growth via open raceway ponds (ORPs) and photobioreactors (PBRs). The scale-up of PBRs is challenged by proper gas exchange and pH maintenance, but has mostly been discouraged by concerns over higher capital expense (Rawat et al. 2013). The latter has often led to pessimism regarding the use of PBRs for large-scale algae production, but recent studies have suggested that the higher productivity resulting from control of growth parameters leads to lower overall costs than those observed with ORPs (Norsker et al. 2011). Economies of scale arising from increasing production will also continue to reduce costs, and Acién et al. (2012) observed that increasing production by a factor of 50 decreased production cost per gallon by a factor of 5. Other studies have found that systems making use of a hybridized approach, involving both PBRs (for inoculation) and ORPs (for large-scale production and lipid accumulation) (Brennan and Owende 2010; Quinn et al. 2013), have the potential to greatly improve the cost competitiveness of algal biofuels. In all cases, however, the costs of inputs weigh heavily in the determination of overall production costs. Consequently, there is considerable interest in the impacts of co-location scenarios that involve low cost (or no cost) opportunities to acquire CO_2_ (e.g., via flue gas from power generation) as well as water and nutrients (e.g., municipal or agricultural wastewater). Access to these low-cost inputs can combine to decrease production costs by more than 50 % (Fernandez et al. 2012); however, physiological issues related to growth on these nutrient sources (Kirrolia et al. 2013) and co-location challenges (Liu et al. 2013) continue to require further research.

Life cycle analysis has been, and continues to be, successfully utilized to identify optimal algal biofuel production pathways. Ongoing refinement and application of this analytical technique can lead to advances that will guide future research toward a better understanding of the implications of many important choices, and thereby promote the development of more cost-effective and environmentally benign biofuel production processes. LCA has successfully identified synergies and tradeoffs between the growth stage and other parts of the production process, and results suggest that parallel research efforts involving both experimental research and life-cycle modeling can be effective (Collet et al. 2013).

## Conclusion

With the reinvigorated interest in alternative fuels, microalgae provide one option that will likely contribute to an overall plan for biomass, biochemical, and biofuel production in a more sustainable and efficient manner. Given the typical ratio of C/N/P in microalgal biomass (C_106_/N_16_/P_1_), much of the research has focused on N and P (P to a lesser extent) and these two elements are linked in different ways to C through resource allocation at the cellular, population, and community levels. In addition, the supply of C either as CO_2_ or bicarbonate at critical times in the growth cycle can significantly improve lipid and biomass productivity. Micronutrients also play a role in cellular responses and activity, and Si and Fe need to be further studied with respect to C/N/P ratios and the allocation of C into desired compounds (e.g., lipids). Diatoms have potential for important contributions to lipid and biomass production but are less studied than the green algae. Many of the nutrient-deprived states have been studied with monocultures (or nearly axenic) as suspended cultures, and regardless of the systems used (e.g., closed reactors vs. open ponds), communities will assemble with different characteristics of stability, resiliency, and productivity. In addition, biofilms will likely develop, and may even be desired for the traits of accumulated biomass that can provide advantages for harvesting.

Moreover, while not directly covered in this mini-review, other resources/conditions will affect the cultivation of microalgae and include water, climate (e.g., light and temperature), land, and location (i.e., geography). Water will be essential for any biological process, and the water recycle will be crucial as many parts of the globe become increasingly stressed for potable water. Light is obviously an important parameter for phototrophs, and is inherently related to temperature as the need for light energy and heat-regulation scale at different proportions. Land is an essential commodity whether bioreactors or ponds are used and should not compete with agricultural needs. The location of growth and processing facilities are crucial aspects to be considered via LCA both for economic implications as well as the biology/ecology (e.g., biogeography) that can differ from region to region. Therefore, targeted science and engineering research is needed to better inform life cycle analyses and process design to maximize productivity, efficiency, and cost ratios.
